# Is having a moderate or low history, electrocardiogram, age, risk factors, troponin risk score a handicap for long-term mortality?

**DOI:** 10.1590/1806-9282.20230745

**Published:** 2024-05-03

**Authors:** Ertan Sonmez, Bahadır Taslidere, Mustafa Alper Deniz, Hande Kahraman, Abuzer Ozkan, Bedia Gulen

**Affiliations:** 1 Malatya Education and Research Hospital, Department of Emergency Medicine – Malatya, Turkey.; 2 Bezmialem Vakif University, School of Medicine, Medical Faculty, Department of Emergency Medicine – İstanbul, Turkey.; 3 Umraniye Training and Research Hospital, Department of Emergency Medicine – İstanbul, Turkey.; 4 Medipol University, School of Medicine, Medical Faculty, Department of Emergency Medicine – İstanbul, Turkey.

**Keywords:** Heart disease, Acute coronary syndrome

## Abstract

**OBJECTIVE:**

History, electrocardiogram, age, risk factors, troponin risk score and troponin level follow-up are used to safely discharge low-risk patients with suspected non-ST elevation acute coronary syndrome from the emergency department for a 1-month period. We aimed to comprehensively investigate the 6-month mortality of patients with the history, electrocardiogram, age, risk factors, troponin risk score.

**METHODS:**

A total of 949 non-ST elevation acute coronary syndrome patients admitted to the emergency department from 01.01.2019 to 01.10.2019 were included in this retrospective study. History, electrocardiogram, age, risk factors, troponin scores of all patients were calculated by two emergency clinicians and a cardiologist. We compared the 6-month mortality of the groups.

**RESULTS:**

The mean age of the patients was 67.9 (56.4–79) years; 57.3% were male and 42.7% were female. Six-month mortality was significantly lower in the high-risk history, electrocardiogram, age, risk factors, troponin score group than in the low- and moderate-risk groups: 11/80 (12.1%), 58/206 (22%), and 150/444 (25.3%), respectively (p=0.019).

**CONCLUSION:**

Patients with high history, electrocardiogram, age, risk factors, troponin risk scores are generally treated with coronary angioplasty as soon as possible. We found that the mortality rate of this group of patients was lower in the long term compared with others. Efforts are also needed to reduce the mortality of moderate and low-risk patients. Further studies are needed on the factors affecting the 6-month mortality of moderate and low-risk acute coronary syndrome patients.

## INTRODUCTION

Many studies have investigated the strategies that can be developed to prevent acute coronary syndrome (ACS) from being overlooked in patients admitted to the emergency department (ED) with chest pain or cardiac symptoms^1,2^. Testing of high-sensitivity troponin I (hs-TnI) levels and the use of certain risk scores [TIMI, GRACE, history, electrocardiogram, age, risk factors, troponin (HEART), EDACS] greatly reduced this outcome^2^. Patients with a low-risk score are safely discharged from the hospital early, while those with a high-risk score are usually treated with appropriate treatment methods during coroner angiography^3,4^.

Major adverse cardiac event (MACE) was defined as definite or probable nonfatal myocardial infarction (MI), nonfatal stroke, or mortality caused by cardiovascular diseases. MACE is increasingly used in randomized controlled trials and observational studies^5^. MACE rates in the first 1–1.5 months after discharge are <2%^6-8^ and <3.3%^9^, which is a satisfactory level. Risk scores have been extensively studied using the 1-month MACE rates^7,9^.

We did not encounter any studies comparing the high-risk group with other groups in terms of their 6-month mortality. In this study, we wanted to investigate the positive effects of interventional treatments on mortality in patients with high HEART risk scores and the long-term outcomes of moderate- and low-risk patients who were discharged safely in the short term.

## METHODS

This is a retrospective cohort study conducted with data collected in the ED of a tertiary university hospital with a monthly admission of 24,000–30,000 patients. The study was approved by the Bezmialem Vakif University Ethics Committee (number E-54022451-050.05.04-42848 and decision number 2021/386). The data were obtained from the hospital's patient clinical information system (Nucleus MBS). In this study, 949 patients admitted to the ED from 01.01.2019 to 01.10.2019 were diagnosed with non-ST elevation ACS (NSTE-ACS). The strengthening the reporting of observational studies in epidemiology (STROBE) checklist was used to form this study^10^.

### Definitions and variables

Clinical presentation of NSTE-ACS was anginal pain [with prolonged (0.20 min) anginal pain at rest, new onset (de novo) angina (class II or III of the Canadian Cardiovascular Society classification), recent destabilization of previously stable angina with at least Canadian Cardiovascular Society Class III angina characteristics (crescendo angina), and post-MI angina]^11^.

We evaluated the outcome retrospectively by examining the citizen information system (E-DEVLET) and evaluated them for significant differences in 6-month mortality. The data were classified according to the outcome of the patients: diagnosed with NSTE-ACS, discharged from the ED [emergency outpatient (EO)], treated in the intensive care unit (ICU), or treated in other clinics (OC). The HEART scores of all patients were calculated by two emergency clinicians and a cardiologist. The HEART score classifies NSTE-ACS as low (0–3), moderate (4–6), or high (7–10) risk (Table 1). We analyzed patients diagnosed as NSTE-ACS checking mortality at 6 months. All assessments were of troponin I and were performed by the hospital laboratory using a chemiluminescent microparticle immunoassay (ARCHITECT STAT High Sensitivity Troponin-I Assay, Abbot Laboratories, USA) (99th percentile normal concentration <34.2 pg/mL).

**Table 1:** The history, electrocardiogram, age, risk factors, troponin score.

HEART score		
History	Highly suspicious	2
	Moderately suspicious	1
	Slightly suspicious	0
ECG	Significant ST depression	2
	Non-specific repolarization disturbance	1
	Normal	0
Age	≥65 years	2
	>45 to <65 years	1
	≤45 years	0
Risk factors	≥3 risk factors,^a^ or history of atherosclerotic disease^b^	2
	1 or 2 risk factors	1
	No risk factors known	0
Troponin	≥3× normal limit	2
	>1 to <3× normal limit	1
	≤normal limit	0

a Risk factors: diabetes mellitus, smoking, hypertension, hypercholesterolemia, family history of coronary artery disease, and obesity (body mass index >30).

b History of atherosclerotic disease: coronary revascularization, myocardial infarction, stroke, and peripheral arterial disease.

### Statistical analysis

The sample size was calculated with a two-tailed alpha of 0.05, a two-tailed beta of 0.1, an estimated AUC of 0.860, and a ratio of patients with a negative–positive outcome of 0.0526. Accordingly, it was determined that approximately 88 patients, with at least 83 living and 5 died, needed to be collected. The Shapiro-Wilk test was used in the analysis of the normality of data. The data did not follow a normal distribution. Categorical data were presented as numbers (%) and compared with the chi-square test. Quantitative variables were presented as median and interquartile range (25–75th percentile) values and then compared for the three groups using the Kruskal-Wallis test. To compare subgroups, we conducted pairwise comparisons using the Dwass-Steel-Critchlow-Fligner method. Statistical significance was determined as p=0.05 for all cases. To investigate mechanisms that potentially underlie the relationship between HEART score and mortality, age, diagnosis of chronic diseases, ECG, complaint analysis, and risk factors were considered. All data used for the calculation of statistical values were anonymized before being provided to the researchers.

## RESULTS

As the records of all patients are registered in the E-DEVLET and death notification is mandatory, no case is excluded due to death information. The mean age of the patients was 67.9 (56.4–79) years, with 57.3% male and 42.7% female. The complaints of the patients were as follows: cardiac chest pain 211 (22.2%), non-cardiac chest pain 259 (27.3%), and ACS equivalent symptoms 479 (50.5%) (i.e., fatigue, sweating, and fainting). The HEART score distribution was 264 (27.8%) in the low-risk group, 594 (62.6%) in the moderate-risk group, and 91 (9.6%) in the high-risk group (Table 2).

**Table 2:** Distribution of history, electrocardiogram, age, risk factors, troponin risk groups of patients by age, gender, complaints, and 6-month mortality.

HEART groups						
		Low	Moderate	High	Total	p
		n (%)	n (%)	n (%)	n (%)	
Age (years)	Median (IQR)	59 (45.2–71.6)	70.8 (59.4–80.6)	70.2 (61.4–76.9)	67.9 (56.4–79.0)	<0.001
Sex	Male	160 (60.6)	332 (55.9)	52 (57.1)	544 (57.3)	0.436
	Female	104 (39.4)	262 (44.1)	39 (42.9)	405 (42.7)	
Complaints	Cardiac chest pain	11 (4.2)	147 (24.7)	53 (58.2)	211 (22.2)	<0.001
	Noncardiac chest pain	58 (22.0)	174 (29.3)	27 (29.7)	259 (27.3)	
	ACS equivalent symptoms	195 (73.9)	273 (46.0)	11 (12.1)	479 (50.5)	
Mortality	No	206 (78.0)	444 (74.7)	80 (87.9)	730 (76.9)	0.019
	Yes	58 (22.0)	150 (25.3)	11 (12.1)	219 (23.1)	
	p*	0.100^a^	0.555^b^	0.016^c^		
	Total	264 (27.8)	594 (62.6)	91 (9.6)		

ACS: acute coronary syndromes; IQR: interquartile range.

* Mann-Whitney U test; Kruskal-Wallis test; chi-square test; Dwass-Steel-Critchlow-Fligner pairwise comparisons test.

a Low and high comparison;

b low and moderate comparison;

c moderate and high comparison.

We classified the patients into four categories according to the outcome: 500 (52.7%) were EO, 251 (26.4%) were treated in the coronary unit (CU), 100 (10.5%) were treated in the ICU, and 98 (10.3%) were treated in OC. The distribution of 6-month mortality according to outcome was as follows. Of the 251 patients treated at the CU, 22 (2%) died. Of the 100 patients whose troponin elevation was associated with Type 2 MI in the ICU, 67 (7%) died. A total of 98 patients whose troponin levels were associated with other causes were treated in OC with various diagnoses, of whom 36 (4%) died. Patients who were not diagnosed with MI or other serious diseases and were not treated by hospitalization (patients who did not show an increase or decrease in troponin follow-up and had chronic diseases) were EO. Of these 500 patients, 94 (10%) died (Table 3).

**Table 3:** Six-month mortality of patients according to outcomes.

Outcome of patient groups			
	n (groups)(%)	Mortality	n (%)
Emergency outpatient	500 (52.7)	No	406 (43)
		Yes	94 (10)
Coronary unit	251 (26.4)	No	229 (24)
		Yes	22 (2)
Intensive care	100 (10.5)	No	33 (3)
		Yes	67 (7)
Other clinics	98 (10.3)	No	62 (7)
		Yes	36 (4)
Total	949 (100)		949 (100)

When we compared the mortality rates of all patients between the HEART groups, we found that at least two groups had statistically significant differences (p=0.019). There were 58 (22%) mortalities in the low-risk group, 150 (25.3%) mortalities in the moderate-risk group, and 11 (12.1%) mortalities in the high-risk group. Post hoc analysis found that the moderate- and high-risk HEART groups’ mortality is different (p=0.016) (Table 2).

## DISCUSSION

In this study, we showed the relationship of HEART risk score with the 6-month mortality of patients.

Patients who are considered low risk in heart score have low MACE (1–1.5 months) rates^7-9^. When used in combination with serial troponin measurements, the HEART score allows more patients to be discharged early and safely, limits heart test rates, and reduces hospital stays^12^. Most studies have used classical troponin when calculating the HEART score^13-15^. However, serial measurement of conventional troponin provides limited benefit in low-risk HEART score patients^16^. The HEART score consists of age, risk factors, history, ECG, and troponin level. Low-risk patients, defined by a score of 0–3, show a low MACE rate (<2%). This score decreases admission for chest pain by at least 20%, with a negative predictive value for MACE (>99%)^17^. Moderate-risk patients (scoring 4–6) show a 12–16.6% risk of MACE, and high-risk patients (scoring ≥7) have a 50–65% risk of MACE^18^. According to these MACE rates, more interventional treatments and bypasses should be performed in high-risk patients, fewer in moderate-risk patients, and a few in low-risk patients.

We compared 6-month mortality for HEART risk score groups, and the results were interesting. The mortality rate was highest in the moderate-risk group (25.3%) and lowest in the high-risk group (12.1%). There was a significant difference between the medium and high-risk groups (p=0.016) (Table 2). One reason for this may be that there is less invasive examination and treatment in the moderate-risk group. For these, we may recommend a further heart examination. Patients in the moderate- and low-risk groups are usually discharged after being called for a follow-up examination for tests such as outpatient exercise tests, cardiac scintigraphy, and echocardiography. However, a significant portion of these patients do not undergo invasive tests because they do not come to their follow-up examinations on time or because the sensitivity of the tests predicts negative risks in the near future. In this study, we showed that these patients face a significant increase in mortality within 6 months.

Clinicians have difficulty diagnosing NSTE-ACS patients who do not have cardiac chest pain but have ACS-equivalent symptoms if their troponin is high. In these patients, in addition to important diagnoses such as diabetic ketoacidosis, sepsis, pneumonia, shock, acute pancreatitis, and acute renal failure, simpler diagnoses such as minor infection, mild electrolyte disorder, and mild dehydration can be considered. This is observed in the type 2 MI group treated in the ICU, whose troponin elevation is attributed to other important underlying diseases. While the 6-month mortality of type 1 MI patients treated in CU was low (2%), the mortality of type 2 MI patients treated in ICU was high (7%). Additionally, the mortality of patients treated in OC with any diagnosis was also high (4%) (Table 3). According to these results, the mortality of those who receive interventional treatment is better than that of the other groups. The HEART risk score does not score ACS patients in terms of comorbid diseases. In this case, even if the patient's score is calculated as low or moderate risk, their mortality may be high. However, studies are needed to answer the question of how cardiac invasive diagnoses and treatments may contribute to mortality in these patients.

A multicenter prospective study recommends designating a HEART score of 2 or less as the cutoff point not to miss MI in patients considered low risk^19^. The fact that the mortality of EO patients (10%) is higher than that of inpatients (2, 7, and 4%) may be related to this result (Table 3). Given our results, we think that some NSTE-ACS patients may not be given the advanced cardiac evaluation they need. We recommend that these patients undergo further cardiac evaluation after other treatments are completed. In a review written on non-coronary troponin elevation, cardiac examination is recommended if ECG or ischemic findings persist in patients when other pathologies that increase troponin have been treated and eliminated^20^.

## LIMITATIONS

The limitation of the study was that cardiac controls of those with moderate- and low-risk heart scores were performed as outpatients, and we did not know what kind of controls they had.

## CONCLUSION

Invasive treatments are mostly applied to patients in the HEART high-risk group. Mortality rates between risk groups as a result of these treatments have not been compared before. The high-risk group benefited greatly from the heart treatments they received and had low 6-month mortality rates. We believe that studies that will reduce mortality in moderate- and low-risk groups are needed.
